# Occupational stress among nurses in the Ashanti Region of Ghana: a comparative cross-sectional study of government and private hospitals

**DOI:** 10.1186/s12912-026-04365-w

**Published:** 2026-01-27

**Authors:** Benjamin Antwi Boasiako, Joana Apenkwa, Denis Dekugmen Yar, Prince Kwabena Achoo, Crispen Kandatam

**Affiliations:** Department of Public Health Education, Faculty of Environment and Health Education, Akenten Appiah -Menka University of Skills Training Entrepreneurial Development, Mampong, Ghana

**Keywords:** Hospital, Nurse, Occupational stress, Stress, Stressors, Work-related stress

## Abstract

**Background:**

Occupational stress, which negatively affects nurses’ health, job satisfaction, and quality of patient care, is a major challenge in nursing. This study conducted a comparative analysis of occupational stress among nurses in government and private hospitals in the Ashanti Region of Ghana, identifying levels, predictors, effects, and coping strategies.

**Methodology:**

A hospital-based comparative cross-sectional study was conducted among 375 nurses selected through multistage sampling. Data were collected via structured questionnaires covering demographic data, stress levels, influencing factors, effects, and coping mechanisms. The data were analysed with SPSS version 27.0 via descriptive statistics, chi-square tests and logistic regression.

**Results:**

High occupational stress was reported by 51.2% of nurses, with stress levels significantly higher in government hospitals than in private hospitals [AOR = 2.43 (1.04, 5.65), *p* = 0.039]. Marital status, age, rank, and years of experience were significantly associated with stress levels. Key stressors included heavy workload (69.6%), inadequate resources (61.1%), lack of overtime pay (61.6%), and unsatisfactory salaries (64.3%). Nurses in government hospitals were twice as likely to report reduced patient care attitudes [AOR = 1.78 (1.06–3.00), *p* = 0.030]. Emotional stressors such as patient deaths were reported by 81.1% of the nurses. Nurses adopted coping strategies such as time management, hobbies, and peer support.

**Conclusion:**

This study revealed that a significant proportion of nurses in both government and private hospitals in the Ashanti Region experienced high levels of occupational stress. Nurses in government hospitals were more likely to report stress than were those in private hospitals. This imply that nurses’ stress can harm their well-being and patient care. To mitigate stress, hospital management and policymakers should strengthen support systems, ensure fair remuneration, recognize nurses’ efforts, and enforce adequate staffing and resources.

## Introduction

Occupational stress has emerged as a significant global health concern, particularly within professions that demand continuous human interaction and high responsibility such as nursing [1].

The International Labour Organization [2] defines occupational stress as harmful physical and psychological responses that occur when work demands exceed an individual’s capacity to cope. Nursing is consistently among the most stressful occupations worldwide because of the emotional labour, unpredictable workloads, and critical decision-making inherent in the profession [3]. From the literature, work-related stress varies significantly between 9.2% and 75%. In the United Kingdom (UK) alone, research work conducted reported a 68% level of work-related stress as against 75% in Ghana [4–6].

Evidence indicates that occupational stress among nurses has serious implications for health delivery. Studies report that 36.5% of nurses experience burnout, 75.7% report low job satisfaction, and over 36.3% suffer mental health challenges, with stress being significantly associated with lower patient safety, increased errors, and reduced quality of care [7–9].

In many low- and middle-income countries (LMICs), including those in sub-Saharan Africa, the burden of occupational stress is further intensified by systemic healthcare challenges [10]. In Ghana, nurses constitute the largest proportion of the healthcare workforce and are central to the delivery of both preventive and curative services. However, the Ghana Health Service (GHS) [11] reports that the majority of nurses work under strenuous conditions characterized by insufficient staffing levels, limited equipment, and overwhelming patient numbers.

The number of nurses to patients in Ashanti Region has increased tremendously from 1:450 in 2017 to 1: 605 in 2022 [11]. This has further exacerbated the plight of the nurses working in the Ashanti Region.

The Ashanti Region, which is a major healthcare hub in Ghana, hosts a mix of government and private hospitals where nurses operate under varying conditions. Government hospitals often experience overcrowding, limited resources, and high patient-to-nurse ratios, whereas private hospitals may impose strict performance expectations with fewer support mechanisms [12, 13]. Despite these differences, limited comparative research exists on how occupational stress levels and influencing factors differ between these two types of facilities in the region. Without such insights, policy and organizational responses may remain generic, overlooking the distinct needs of nurses in different hospital settings.

Moreover, occupational stress has far-reaching implications not only for nurses’ physical and mental health but also for patient safety and the overall efficiency of the healthcare system [13]. Without addressing stressors and improving coping strategies, the healthcare workforce may experience declining productivity and morale.

Comparative evidence on stress levels, influencing factors, effects, and coping strategies can inform targeted interventions to mitigate stress and promote a healthier work environment. Therefore, this study conducted a comparative analysis of occupational stress among nurses in government and private hospitals in the Ashanti Region.

## Method

### Study design

A hospital-based comparative cross-sectional design was employed to perform a contrastive analysis of occupational stress among nurses working in government and private health facilities in the Ashanti Region. The study was reported in accordance with the Strengthening the Reporting of Observational Studies in Epidemiology (STROBE) guidelines for cross-sectional studies. The study was undertaken over an eight-month period, conducted between November 2024 and July 2025.

The study included registered nurses aged 18 years and above who had been employed in either government or private hospitals in the Ashanti Region for more than one year and consented to the study. However, nurses on extended leave during the study period, such as maternity or sick leave, were omitted to ensure the sample reflected nurses actively engaged in clinical practice.

### Sampling technique

The multistage sampling technique was used to select 375 nurse respondents. Sample size was estimated using Yamane’s formula. In the first stage, the Ashanti Region was clustered into four geographical zones based on the 43 administrative districts. Using simple random sampling, one district was selected from each zone: Mampong Municipal (Zone A), Adansi South District (Zone B), Kumasi Metropolis (Zone C), and Asante Akim South Municipal (Zone D). In the second stage, both government and private hospitals were selected from these districts. Purposive sampling was used to select government hospitals where only one facility existed in the district, whereas simple random sampling was used where multiple hospitals were present. For private hospitals, simple random sampling was applied to select two facilities from each district, resulting in eight private hospitals.

In the final stage, a list of eligible nurses from the selected hospitals was obtained from hospital administrators. Simple random sampling was then used to select nurses who met the inclusion criteria. This approach ensured representation from both government and private hospitals while minimizing selection bias.

#### Each nurse was selected once and independently

The following government hospitals were sampled for the study: Mampong District Hospital, New Edubiase Government Hospital, and Juaso District Hospital. Additionally, Calvary Health Service, Quality Health Care Hospital, Hilltop Maternity Home, Fountain Specialist Hospital, Adiebeba Hospital, Asafo-Agyei Hospital, Stewards Hospital, and First-Class Hospital were the private hospitals selected for the study.

### Data collection tool(s)

Data were collected via a structured, self-administered questionnaire specifically designed to assess occupational stress among nurses. The questionnaire comprises five sections: sociodemographic information (age, sex, marital status, education, years of experience) and occupational stress levels, which are assessed via standardized Likert scale items. Factors influencing occupational stress include workload, resources, the work environment, and management practices; effects of occupational stress, covering aspects such as job performance, health outcomes, and job satisfaction; and coping strategies, capturing the mechanisms nurses use to manage stress.

The items are adapted and modified from the Occupational Stress Index [14] and other validated workplace stress tools [15, 16]. The adaptation involved contextualizing certain items to reflect the experiences of nurses in Ghanaian government and private hospitals. Modifications were also made to include items related to workload, work environment, and lack of promotion, which were identified in the literature as key distributers to occupational stress in the health sector. The modified questionnaire was reviewed by experts in public health and hospital management to establish content validity, and a pilot study was conducted with 30 nurses from a hospital outside the study area but within the Ashanti Region to assess reliability and internal consistency before the main data collection. This pre-test allowed for the identification of ambiguous questions and assessment of the tool’s practicality. Reliability was determined using Cronbach’s alpha, which yielded a value of 0.83, indicating strong internal consistency across the scale items.

#### Each item can be rated on a 5-point likert scale

1 = Strongly Disagree; 2 = Disagree; 3 = Neutral 4 = agree; 5 = Strongly Agree.

The instrument included closed-ended to facilitate quantitative analysis.

Confidentiality was assured by assigning unique identification codes rather than names, and all collected data were securely stored with restricted access limited to the research team.

### Statistical analysis

The data were analysed via both descriptive and inferential statistical methods. Descriptive statistics, including frequencies, percentages, means, and standard deviations, were computed to summarize participants’ sociodemographic characteristics, stress levels, factors influencing stress, effects of stress, and coping strategies. Chi-square tests were used to examine associations between categorical variables such as hospital type (government or private) and levels of occupational stress. Furthermore, binary logistic regression was conducted to identify predictors of high occupational stress while adjusting for potential confounders such as age, sex, and years of experience. A p -value of less than 0.05 was considered statistically significant, and all analyses were carried out at the 95% confidence level.

## Results

### Demographic characteristics of the respondents

In Table 1, the majority (56.8%) of the respondents were female, 47.7% were single, 38.7% were aged 20–29 years, 38.9% were ranked as Principal Nurse Officer (PNO)/ Chief Nursing Officer (CNO), 50.4% worked in government hospitals, 49.3% had less than 5 years of experience, 62.9% worked 7–9 h per day, and 52.3% reported doing extra work beyond their regular duties.

**Table 1:** Demographic characteristics

Demographic Characteristics	Frequency (*N* = 375)	Percentage %
**Gender**		
Male	162	43.2
Female	213	56.8
**Marital status**		
Single	179	47.7
Widowed/widower	18	4.8
Married	178	47.5
**Age range in years**		
20–29	145	38.7
30–39	89	23.7
40 and above	141	37.6
**Rank**		
SN/ SSN	115	30.7
NO / SNO	114	30.4
PNO / CNO	146	38.9
**Type of Health facility**		
Government hospital	189	50.4
Private hospital	186	49.6
**Number of years of experience**		
< 5 years	185	49.3
5–10 years	119	31.7
> 10 years	71	18.9
**Working hours per a day**		
1–6 h	57	15.2
7–9 h	236	62.9
> 9 h	82	21.9
**Do you do any extra work**		
Yes	196	52.3
No	179	47.7

(Data source: Field survey, 2025)

### Level of occupational stress among nurses in both government and private hospitals

As shown in Table 2, the majority (31.7%) of nurses agreed that they often feel overwhelmed by their work (*p* < 0.001). Approximately 26.4% remained neutral about feeling tired in the morning and dreading work. The highest percentage (26.7%) agreed that they felt drained at the end of the workday. Regarding feeling positive and in control at work, 32.0% agreed. Additionally, 28.0% were neutral about tolerating interruptions at work, whereas 24.8% agreed with feeling indifferent or less caring toward patients. Most nurses (26.1%) agreed that there is a high stress level in their institution, and 27.7% remained neutral in worrying about panicking or making mistakes at work.

Table 3 presents the comparison of occupational stress indicators among nurses in government and private hospitals. A significant association was found between the type of hospital and difficulty tolerating interruptions at work (χ²=6.750, *p* = 0.031). Nurses in government hospitals were 8% less likely to report tolerance to interruptions than those in private hospitals were [AOR = 0.92 (0.44–0.98), *p* = 0.043]. Additionally, nurses in government hospitals were approximately 2 times more likely to report becoming less caring toward patients than were those in private hospitals [AOR = 1.78 (1.06–3.00), *p* = 0.030]. Furthermore, high stress levels were more than 2 times more common among nurses in government hospitals than among those in private facilities [AOR = 2.43 (1.04, 5.65), *p* = 0.039].

Table 4 shows the associations between demographic characteristics and occupational stress among nurses. Marital status, age, rank and years of experience were significantly associated with occupational stress (χ²=7.496, p = 0.024), (χ²=9.519, p = 0.009), (χ²=10.642, p = 0.005), and (χ²=13.438, p = 0.001), respectively. Compared with their counterparts’, married nurses were approximately 3 times more likely to experience high stress [AOR = 2.81 (1.13, 7.01), *p* = 0.027]. Older nurses (aged 40 years and above) were 2 times more likely to report high stress levels than younger nurses were [AOR = 2.13 (1.08–4.42), *p* = 0.041]. Compared with Staff Nurse (SN) / Senior Staff Nurse (SSN), PNO / NO were 57% less likely to experience high stress [AOR = 0.43 (0.07–0.86), *p* = 0.044]. Nurses with more than 10 years of experience were approximately 2 times more likely to experience high stress than those with less than 10 years of experience [AOR = 1.99 (1.01–3.90), *p* = 0.045].

**Table 2:** Levels of occupational stress among nurses in both government and private hospitals

Variables	Responses					*P* value
	Strongly Agree (%)	Agree (%)	Neutral (%)	Disagree (%)	Strongly disagree (%)	
I often feel overwhelmed by my work	80 (21.3)	**119 (31.7)**	98 (26.1)	51 (13.6)	27 (7.2)	**< 0.001**
I feel tired in the morning and dread going to work	62 (16.5)	96 (25.6)	**99 (26.4)**	85 (22.7)	33 (8.8)	**< 0.001**
I feel drained at the end of my workday	96 (25.6)	**100 (26.7)**	85 (22.7)	70 (18.7)	24 (6.4)	**< 0.001**
I feel positive, energetic, and in control at work	66 (17.6)	**120 (32.0)**	106 (28.3)	59 (15.7)	24 (6.4)	**< 0.001**
I find it hard to tolerate interruptions at work	56 (14.9)	83 (22.1)	**105 (28.0)**	94 (25.1)	37 (9.9)	**< 0.001**
I have become indifferent or less caring toward patients	59 (15.7)	**93 (24.8)**	84 (22.4)	84 (22.4)	55 (14.7)	**0.004**
There is a high stress level in my institution	96 (25.6)	**98 (26.1)**	96 (25.6)	56 (14.9)	29 (7.7)	**< 0.001**
I worry about panicking and making mistakes at work	83 (22.1)	82 (21.9)	**104 (27.7)**	61 (16.3)	45 (12.0)	**< 0.001**

(Data source: Field survey, 2025)

**Table 3:** Chi-square and logistic regression analyses of the level of occupational stress between the government and private hospitals

Level of Occupational Stress	Type of Hospital		X^2^ (*P* value)	AOR (95% CI) *P* value
	Government (%)	Private (%)		
**I often feel overwhelmed by my work**				
Yes	87(43.7)	112(56.3)	**7.572(0.006)**	1.38 (0.86, 2.21) 0.185
No	102(58.0)	74(42.0)		**Ref**:
**I feel tired in the morning and dread going to work**				
Yes	64(40.5)	94(59.5)	**10.691(< 0.001)**	1.45 (0.87, 2.42) 0.154
No	125(57.6)	92(42.4)		Ref:
**I feel drained at the end of my workday**				
Yes	89(45.4)	107(54.6)	**4.093(0.043)**	0.79 (0.46, 1.36) 0.398
No	100(55.9)	79(44.1)		Ref
**I feel positive**,** energetic**,** and in control at work**				
Yes	80(43.3)	106(57.0)	**8.06(0.005)**	1.40(0.86, 2.29) 0.178
No	109(57.7)	80(42.3)		Ref:
**I find it hard to tolerate interruptions at work**				
Yes	61(43.9)	78(56.1)	**6.750(0.031)**	**0.92 (0.44**,** 0.98) 0.043**
No	128(54.2)	108(45.8)		Ref
**Have become less caring toward patients**				
Yes	58(38.2)	94(61.8)	**15.324(< 0.001)**	**1.78 (1.06**,** 3.00) 0.030**
No	131(58.7)	92(41.3)		**Ref**:
**Level of stress among nurses**				
High	88(45.4)	106(54.6)	**6.083(0.043)**	**2.43 (1.04**,** 5.65) 0.039**
Low	101(55.8)	80(44.2)		**Ref**
**I worry about making mistakes at work**				
Yes	68(41.2)	97(58.8)	**9.950(0.002)**	1.31 (0.763, 2.23) 0.330
No	121(57.6)	89(42.4)		**Ref**:

(Data source: Field survey, 2025)

**Table 4:** Chi-square analysis and logistic regression of the associations between demographic characteristics and level of occupational stress among nurses

Demographics	Level of stress among nurses		X^2^ (*P* value)	AOR (95% CI) *P* value
	High (%)	Low (%)		
Male	94 (58.0)	68 (42.0)	4.521(0.33)	0.69 (0.44, 1.07) 0.098
Female	100(46.9)	113(53.1)		**Ref**:
**Marital status**				
Single	83(46.4)	96 (53.6)	**7.496(0.024)**	Ref:
Widowed/widower	14(77.8)	4(22.2)		0.95 (0.54, 1.67) 0.858
Married	97(54.5)	81(45.5)		**2.81 (1.13**,** 7.01) 0.027**
**Age**				
20–29	62(42)	83(57.2)	**9.519(0.009)**	Ref:
30–39	46(51.7)	43(48.3)		1.43 (0.68, 3.04) 0.347
40 and above	86(61.0)	55(39.0)		**2.13 (1.08**,** 4.42) 0.041**
**Rank**				
SN / SSN	46(40.0)	69(60.0)	**10.642(0.005)**	**Ref**:
NO / SNO	60(52.6)	54(47.4)		1.40 (0.70, 2.77) 0.341
PNO / NO	88(60.3)	58(39.7)		**0.43 (0.07**,** 0.86) 0.044**
**Number of years of experience**				
< 5 years	83(44.9)	102(55.1)	**13.438(0.001)**	**Ref**:
5–10 years	61(51.3)	58(48.7)		1.76 (0.78 ,3.94) 0.168
> 10 years	50(70.4)	21(29.6)		**1.99 (1.01**,** 3.90) 0.045**
**Working hours per a day**				
1–6 h	27(47.4)	30(52.6)	2.117(0.347)	1.1 (0.53, 2.23) 0.806
7–9 h	119(50.4)	117(49.6)		1.2 (0.70, 2.06) 0.507
> 9 h	48(58.5)	34 (41.5)		**Ref**:
**Do you do any extra work**				
Yes	102 (52.0)	94 (48.0)	0.016 (0.901)	1.2 (0.79, 1.92) 0.361
No	92 (51.4)	87 (48.6)		**Ref**:

(Data source: Field survey, 2025)

### Predictors of occupational stress among nurses in both government and private hospitals

As shown in Table 5, most nurses (81.1%) indicated that emotional stress, such as patient deaths, affects them at work, the majority (61.1%) reported working with inadequate resources, and 70.9% confirmed that qualified staff are available when needed. Approximately 61.9% stated that they are supervised by senior staff, 58.1% face family-related challenges, 56.0% said that they are free to choose their work methods, and 69.6% reported having a heavy workload per shift. More than half (58.4%) noted that they were recognized for good work, but 61.6% reported not being paid for overtime. Additionally, 48.3% expressed dissatisfaction with their colleagues’ attitudes, and 64.3% were not satisfied with their salary.

Table 6 examines the predictors of occupational stress among nurses in government and private hospitals. Recognition of good work and payment for overtime were significantly associated with occupational stress (χ²=5.73, *p* = 0.030 and χ²=5.04, *p* = 0.025, respectively). Nurses who were recognized for good work were 43% less likely to experience occupational stress than those who were not recognized [AOR = 0.57 (0.33,0.98), *p* = 0.042]. Additionally, nurses who received overtime pay were 54% less likely to report stress than those who were not paid for extra work [AOR = 0.46 (0.22, 0.94), *p* = 0.033].

Table 7 shows the associations between demographic characteristics and salary satisfaction among nurses. Marital status, rank, working hours per day, and engagement in extra work were significantly associated with salary satisfaction (χ²=28.32, *p* = 0.000), (χ²=16.93, *p* = 0.000), (χ²=10.21, *p* = 0.006), and (χ²=4.69, *p* = 0.030), respectively. Compared with married nurses, single nurses were 55% more likely to be satisfied with their salary [AOR = 0.45 (0.25–0.84), *p* = 0.011]. Compared with PNO/ CNO, Nurse Officers (NO) / Senior Nurse Officers (SNO) were more than 2 times more likely to be dissatisfied with their salary [AOR = 2.62 (1.31–5.23), *p* = 0.006]. Nurses working 7–9 h per day were 76% more likely to be satisfied with their salary than those working more than 9 h [AOR = 1.76 (1.03, 3.01), *p* = 0.037]. Nurses who did extra work were nearly 2 times more likely to be dissatisfied with their salary than those who did not [AOR = 1.84 (1.41–3.25), *p* = 0.032].

**Table 5:** Predictors of occupational stress among nurses

Variables	Frequency (*N* = 375)	Percentage %
**Do patient deaths affect you at work**		
Yes	304	81.1
No	71	18.9
**Do you work with inadequate resources**		
Yes	229	61.1
No	146	38.9
**Are qualified staff available when needed**		
Yes	266	70.9
No	109	29.1
**Are you supervised by senior staff**		
Yes	232	61.9
No	143	38.1
**Do you face family-related challenges**		
Yes	218	58.1
No	157	41.9
**Are you free to choose your work methods**		
Yes	210	56.0
No	165	44.0
**Is your workload heavy per shift**		
Yes	261	69.6
No	114	30.4
**Are you recognized for good work**		
Yes	219	58.4
No	156	41.6
Are you paid for overtime work		
Yes	144	38.4
No	231	61.6
**Are you dissatisfied with colleagues’ attitudes**		
Yes	181	48.3
No	194	51.7
**Are you satisfied with your salary**		
Yes	134	35.7
No	241	64.3

(Data source: Field survey, 2025)

**Table 6:** Chi-square analysis and logistic regression of the predictors of occupational stress among nurses in government and private hospitals

Predictors of Occupational Stress	Type of Hospital		X^2^ (*P* value)	AOR (95% CI) *P* value
	Government (%)	Private (%)		
**Emotion/Patient deaths affect your work**				
Yes	152 (50.0)	152 (50.50)	0.10 (0.749)	0.95 (0.54, 1.68) 0.853
No	37 (52.1)	34 (47.9)		**Ref**:
**Work with inadequate resource**				
Yes	117 (51.1)	112 (48.9)	0.11 (0.737)	0.86 (0.54, 1.36) 0.520
No	72 (49.3)	74 (50.7)		**Ref**:
**Qualify staff available when needed**				
Yes	129 (48.5)	137 (51.5)	1.33 (0.249)	1.12 (0.68, 1.80) 0.685
No	60 (55.0)	49 (45.0)		**Ref**:
**Workload heavy per shift**				
Yes	127 (48.7)	134 (51.3)	1.04 (0.308)	1.31 (0.82, 2.10) 0.257
No	62 (54.4)	52 (45.6)		**Ref**:
**You are recognized for good work**				
Yes	100 (45.7)	119 (54.3)	**5.73 (0.030)**	**0.57 (0.33**,**0.98) 0.042**
No	89 (57.1)	67 (42.9)		Ref:
**Are you paid overtime work**				
Yes	62 (43.1)	82 (56.9)	**5.04 (0.025)**	**0.46 (0.22**,** 0.94) 0.033**
No	127 (55.0)	104 (45.0)		**Ref**:
**Satisfied with your salary**				
Satisfied	59 (44.0)	75 (56.0)	3.39 (0.066)	1.57 (0.98, 2.53) 0.063
Not satisfied	130 (53.9)	111 (46.1)		**Ref**:

(Data source: Field survey, 2025)

**Table 7:** Chi-square analysis and logistic regression of the association between demographic characteristics and salary satisfaction among nurses in government and private hospitals

Demographics	Satisfied with salary		X^2^ (*P* value)	AOR (95% CI) *P* value
	Satisfied (%)	Not Satisfied (%)		
**Male**	49 (30.2)	113 (69.8)	3.74 (0.053)	1.48 (0.91, 2.40) 0.117
Female	85 (39.9)	128 (60.1)		**Ref**:
**Marital status**				
Single	87 (48.6)	92 (51.4)	**28.32 (0.000)**	**0.45 (0.25**,** 0.84) 0.011**
Widowed/widower	8 (44.4)	10 (55.6)		**0.33 (0.12**,** 0.97) 0.044**
Married	39 (21.9)	139 (78.1)		**Ref**:
**Age**				
20–29	73 (50.3)	72 (49.7)	**23.52 (0.000)**	0.50 (0.22, 1.15) 0.103
30–39	28 (31.5)	61 (68.5)		0.62 (0.31, 1.26) 0.186
40 and above	33 (23.4)	108 (76.6)		Ref:
**Rank**				
SN / SSN	57 (49.6)	58 (50.4)	**16.93 (0.000)**	1.55 (0.74, 3.28) 0.248
NO / SNO	27 (23.7)	87 (76.3)		**2.62 (1.31**,** 5.23) 0.006**
PNO / CNO	50 (34.2)	96 (65.8)		**Ref**:
**Number of years of experience**				
< 5 years	85 (45.9)	100 (54.1)	**16.86 (0.000)**	0.78 (0.32, 1.91) 0.585
5–10 years	29 (24.4)	90 (75.6)		1.12 (0.53, 2.38) 0.771
> 10 years	20 (28.2)	51 (71.8)		**Ref**:
**Working hours per a day**				
1–6 h	31 (54.4)	26 (45.6)	**10.21 (0.006)**	0.53 (0.25, 1.15) 0.108
7–9 h	77 (32.6)	159 (67.4)		**1.76 (1.03**,** 3.01) 0.037**
> 9 h	26 (31.7)	56 (68.3)		**Ref**:
**Do you do any extra work**				
Yes	60 (30.6)	136 (69.4)	**4.69 (0.030)**	**1.84 (1.41**,** 3.25) 0.032**
No	74 (41.3)	105 (58.7)		**Ref**:

(Data source: Field survey, 2025)

### Effects of occupational stress among nurses in both government and private hospitals

As shown in Table 8, the majority (81.9%) of nurses reported experiencing physical health problems such as body pain, 36.3% had mental health challenges, most respondents (59.2%) agreed that patient care has decreased in their facility because of stress, 45.3% reported high nurse absenteeism in their facility, and 53.9% stated that staff turnover is high.

**Table 8:** Effects of occupational stress among nurses

Variables	Frequency (*N* = 375)	Percentage %
**Experience physical health problems like body pain**		
Yes	307	81.9
No	68	18.1
**Do you have mental health challenges**		
Yes	136	36.3
No	239	63.7
**Has patient care reduced in your facility**		
Yes	222	59.2
No	153	40.8
**Is nurse absenteeism high in your facility**		
Yes	170	45.3
No	205	54.7
**Is staff turnover high in your facility**		
Yes	202	53.9
No	173	46.1

(Data source: Field survey, 2025)

**Table 9:** Effects of occupational stress among nurses in government and private hospitals

Effects of Occupational Stress	Type of Hospital		X^2^ (*P* value)
	Government (%)	Private (%)	
**Experience physical problem like body pain**			
Yes	154 (50.2)	153 (49.8)	0.04 (0.845)
No	35 (51.5)	33 (48.5)	
**Have mental challenge**			
Yes	66 (48.5)	70 (51.5)	0.30 (0.585)
No	123 (51.5)	116 (48.5)	
**Has patient care reduced in your facility**			
Yes	108 (48.6)	114 (51.4)	0.67 (0.414)
No	81 (52.9)	72 (47.1)	
**Nurses’ absenteeism is high in my facility**			
Yes	84 (49.4)	86 (50.6)	0.12 (0.727)
No	105 (51.2)	100 (48.8)	
**High staff turnover in my facility**			
Yes	96 (47.5)	106 (52.5)	1.45 (0.229)
No	93 (53.8)	80 (46.2)	

(Data source: Field survey, 2025)

Table 9 examines the effects of occupational stress among nurses in government and private hospitals. There were no associations between any of the effects (such as physical problems such as body pain, mental challenges, reduced patient care, high absenteeism, or high staff turnover) and the type of hospital.

### Coping strategies adopted by nurses both governmentally and privately hospitals

As shown in Table 10, the majority (37.3%) of nurses agreed that they use positive self-talk to cope with stress at work (*p* < 0.001). Approximately 34.1% of the participants remained neutral in talking to family, friends, or colleagues for stress relief. The majority (30.9%) agreed that they tried to control their emotions and reactions when they were under stress. Additionally, 28.8% were neutral about believing that they are not responsible for stressful situations. Most participants (35.7%) agreed that they reward themselves to relieve stress, whereas 25.1% agreed that they avoid or withdraw from stressful work situations.

Table 11 examines the coping strategies adopted by nurses in government and private hospitals. Seeking support from family and friends, rewarding oneself, and avoiding stressful situations were significantly associated with stress coping strategies (χ²=6.39, *p* = 0.011), (χ²=7.50, *p* = 0.019), and (χ²=10.12, *p* = 0.001), respectively. Nurses who sought support from family and friends were 1.4 times more likely to adopt effective coping strategies than those who did not [AOR = 1.40 (1.06–2.89), *p* = 0.021]. Those who rewarded themselves were approximately 2 times more likely to cope effectively with stress than those who did not [AOR = 2.18 (1.01–2.95), *p* = 0.047]. Additionally, nurses who avoided stressful situations were 1.6 times more likely to adopt better coping mechanisms than those who did not [AOR = 1.61 (1.02–2.514), *p* = 0.042].

**Table 10:** Coping strategies adopted by nurses

Variables	Responses					*P* value
	Strongly Agree (%)	Agree (%)	Neutral (%)	Disagree (%)	Strongly disagree (%)	
I use positive self-talk to help me cope with stress at work	80 (21.3)	140 (**37.3**)	89 (23.7)	49 (13.1)	17 (4.5)	**< 0.001**
I talk to family, friends, or colleagues to help manage my stress	49 (13.1)	118 (31.5)	128 (**34.1**)	64 (17.1)	16 (4.3)	**< 0.001**
I try to control my emotions and reactions when under stress	82 (21.9)	116 (**30.9**)	96 (25.6)	55 (14.7)	26 (6.9)	**< 0.001**
I believe I am not responsible for stressful situations at work	60 (16.0)	100 (26.7)	108 (**28.8**)	74 (19.7)	33 (8.8)	**< 0.001**
I reward myself with something I enjoy to relieve stress	69 (18.4)	134 (**35.7**)	94 (25.1)	59 (15.7)	19 (5.1)	**< 0.001**
I avoid or withdraw from stressful work situations	57 (15.2)	94 (**25.1**)	78 (20.8)	75 (20.0)	71 (18.9)	**< 0.001**

(Data source: Field survey, 2025)

**Table 11:** Chi-square analysis and logistic regression of the coping strategies adopted by nurses in government and private hospitals

Coping Strategies of Stress	Type of Hospital		X^2^ (*P* value)	AOR (95% CI) *P* value
	Government (%)	Private (%)		
**Use positive self-talk to cope**				
Yes	101 (45.9)	119 (54.1)	**4.29 (0.038)**	1.37 (0.86, 2.18) 0.190
No	88 (56.8)	67 (43.2)		**Ref**:
**Seek family and friends support to cope**				
Yes	72 (43.1)	95 (56.9)	**6.39 (0.011)**	**1.40 (1.06**,** 2.89) 0.0021**
No	117 (56.3)	91 (43.7)		**Ref**:
**I try to control my emotions**				
Yes	95 (48.0)	103 (52.0)	0.98 (0.321)	0.80 (0.48, 1.34) 0.394
No	94 (53.1)	83 (46.9)		**Ref**:
**I reward myself to relieve stress**				
Yes	91 (44.8)	112 (55.2)	**750 (0.019)**	**2.18 (1.01**,** 4.70) 0.047**
No	98 (57.0)	74 (43.0)		**Ref**:
**I avoid stressful situation**				
Yes	61 (40.4)	90 (59.6)	**10.12 (0.001)**	**1.61 (1.02**,** 2.514) 0.042**
No	128 (57.1)	96 (42.9)		**Ref**:

(Data source: Field survey, 2025)

## Discussion

### Level of occupational stress among nurses in government and private hospitals

The study revealed that about 51% of respondents reported working in a high-stress environment, consistent with findings from Ghana and other LMICs where understaffing, low pay and long shifts are prevalent [17, 18].

The findings revealed a significantly greater level of occupational stress among nurses in government hospitals than among their counterparts in private facilities. This could be attributed to systemic differences in working conditions, such as higher patient loads, administrative bureaucracy, and resource constraints, which are often characteristic of government health facilities [19]. In contrast, private hospitals are often better resourced, more flexible in management practices, and maintain better nurse-to-patient ratios, which can contribute to reduced work-related stress. The implication of this finding is critical, as chronic occupational stress not only impacts the mental and physical health of nurses but also affects the quality of care provided to patients [20]. Therefore, interventions aimed at improving working conditions in government hospitals such as staff redistribution, workload reduction, and better resource allocation are urgently needed to mitigate occupational stress among nurses. Government nurses were less tolerant of interruptions and showed higher emotional exhaustion, consistent with Job Demand – Resource model [21, 22]. The fact that nurses in government hospitals are more likely to become emotionally detached may signal compromised professional quality of life and potentially reduced patient satisfaction.

These findings have several implications for healthcare administrators, policymakers, and stakeholders. First, the moderate levels of stress suggest the need for immediate intervention to prevent escalation to severe burnout and compromised patient care. Strategies such as periodic stress assessments, staff rotation, adequate staffing, and provision of rest periods are necessary. Furthermore, training in stress management, emotional intelligence, and resilience-building should be integrated into institutional capacity-building programs. Policymakers must prioritize nurse welfare and mental health in workforce planning.

### Predictors of occupational stress among nurses in government and private hospitals

The current study identified recognized nurses were 43% less likely, and those paid overtime 54% less likely, to experience stress. This supports Herzberg’s theory that recognition and fair pay reduce stress, as shown in previous studies [23–25]. The implication of this finding is profound; healthcare managers must institutionalize reward systems and enforce equitable remuneration for overtime to promote a supportive work culture and minimize stress among nurses.

Further analysis revealed that salary satisfaction among nurses was significantly associated with several demographic variables. Single nurses were 55% more likely to be satisfied with their salaries than married ones, consistent with Yusof et al. [26], who found out that family obligations increase financial strain among nurses. Hence, this suggests that salary and incentive policies should consider family-oriented benefits or allowances for married nurses to enhance satisfaction and retention.

Rank within the nursing profession also significantly influenced salary satisfaction. Nursing Officers (NO) / Senior Nursing Officer (SNO) were over twice as likely to be dissatisfied with their salaries than Principal Nursing Officer (PNO) / Chief Nursing Officer (CNO), consistent with Dinh [27] who found lower-ranked staff often feel undervalued. This disparity may stem from differences in job security, decision-making power, and income levels associated with higher ranks. To address this, career development opportunities, transparent promotion criteria, and periodic salary reviews for lower-ranked nurses should be prioritized to foster equity and motivation in the workplace. Nurses who do extra duties were nearly twice as likely to be dissatisfied with their salaries, supporting [28] that excessive unpaid workload drives job dissatisfaction. The implication is that reducing unnecessary workload and ensuring that all extra tasks are appropriately compensated can significantly improve salary satisfaction and overall staff morale.

These findings highlight the need for equitable compensation structures, recognition systems, and workload management strategies tailored to the unique needs of nurses across different hospitals and demographic categories.

### Effects of occupational stress among nurses in government and private hospitals

The present study revealed significant physical and mental health effects of occupational stress among nurses working in both government and private hospitals in the Ashanti Region. A majority of the respondents (81.9%) reported experiencing physical health issues such as body pain, suggesting that prolonged standing, heavy workloads, and poor ergonomic conditions are contributing factors. This aligns with findings from studies [29, 30] indicating that musculoskeletal disorders and fatigue are common outcomes of stress among healthcare workers in low-resource settings.

Additionally, 36.3% of nurses admitted to having mental health challenges, a result that is consistent with global research emphasizing the psychological toll of nursing work, particularly in environments with high patient loads, insufficient breaks, and limited psychosocial support [31, 32]. These findings highlight the dual burden of physical and emotional strain among nurses and the urgent need for workplace interventions such as regular health screenings, mental health counselling, and stress management training.

The current study further assessed the effects of occupational stress among nurses in both government and private hospitals and found no significant associations between hospital type and reported stress-related outcomes. This suggests that, regardless of institutional setting, the impact of occupational stress manifests uniformly among nurses, reflecting the demanding nature of the nursing profession itself. The lack of difference also implies that interventions targeting stress effects should not be limited to one type of facility but implemented across both sectors.

Hospital administrators should therefore adopt universal stress-reduction strategies such as mental health support, regular breaks, and employee wellness programs to address the adverse outcomes of occupational stress.

### Coping strategies adopted by nurses in government and private hospitals

The current study explored the coping strategies adopted by nurses in both government and private hospitals in the Ashanti Region to manage occupational stress. A significant proportion of participants (44%) reported discussing stress with others, unlike Western healthcare workers who more often use counselling support [33]. Social interaction helps nurses process workplace stressors, providing both emotional relief and practical advice, regardless of whether they work in public or private settings.

Nurses who practiced self-reward were nearly as likely to cope with stress, supporting self-care and positive reinforcement theories [34]. It highlights the importance of promoting self-care strategies among nurses to enhance their resilience and motivation in the face of work-related challenges.

Nurses who avoided stressful situations were 1.6 times more likely to cope effectively, using a protective strategy to reduce emotional strain [35]. However, prolonged use of avoidance can also lead to unresolved stress and burnout if not balanced with active problem-solving. Therefore, while avoidance can be temporarily beneficial, healthcare institutions should provide structured support systems, such as stress management training and counselling services, to promote long-term psychological resilience.

## Conclusion

The study revealed occupational stress among nurses in both government and private hospitals was high.

Government nurses reported more difficulty handling interruptions and emotional detachment, showing higher work- related stress in the public sector. This imply that nurses’ stress can harm their well-being and patient care. Highlighting the need for organizational support.

Additionally, recognition of good work and overtime pay significantly reduced the likelihood of stress. The findings suggest that recognition and fair overtime pay help mitigate nurses’ stress levels. Occupational stress negatively affected nurses’ physical and mental health. This underscores the urgent need for mental health support systems within healthcare. Common coping strategies: positive self-talk; family /friend support; self-reward; avoiding stress – all linked to lower stress levels. Integrating stress management and healthy coping strategies is key to enhancing nurses’ mental resilience.

The Ministry of Health (MoH) of Ghana should promote policies that enforce fair compensation, including timely payment over time, to reduce financial-related stress among nurses. Facilities should introduce recognition systems and staff appreciation initiatives to motivate healthcare professionals and reduce burnout. Nurses should prioritize self-care and seek support from others to effectively manage occupational stress.

Future studies should adopt longitudinal or mixed-method approaches to examine the long-term impacts of occupational stress and coping strategies among nurses across different regions of Ghana.

This study primarily relied on self-reported data collected through structured questionnaires administered to nurses working in government and private hospitals within the Ashanti Region. This method may have introduced recall bias or social desirability bias, where participants might have underreported or over reported their experiences of occupational stress, its effects, or coping strategies in a manner they believed to be more socially acceptable. To address this, participants were assured of confidentiality and anonymity to encourage honest and objective participation.

Another limitation concerns the geographical focus of the study, which was confined to selected hospitals within the Ashanti Region. While the findings provide valuable insights into occupational stress among nurses in this region, they may not be generalizable to other regions or healthcare settings in Ghana. Additionally, the use of a cross-sectional design limits the ability to establish causal relationships between stress predictors and outcomes. Moreover, the sampling approach may not fully capture the diversity of experiences among all nurses in the region, especially those in smaller or rural health facilities.

Despite these limitations, the study contributes significantly to the understanding of occupational stress among nurses in Ghana and offers practical evidence to inform interventions aimed at improving nurse well-being and performance in both public and private healthcare systems. Moreover, the study fills a critical gap in the literature on occupational stress by providing empirical evidence from nurses in hospital settings, particularly within resource-limited contexts.

## Data Availability

The datasets used and analysed during the current study are available from the corresponding author upon reasonable request.

## Abbreviations

AAMUSTED: Akenten Appiah- Menka University of Skills Training and Entrepreneurial Development
CNO: Chief Nursing Officer
GHS: Ghana Health Service
ILO: International Labour Organization
LMICs: Low- and middle- income countries
MoH: Ministry of Health
NO: Nursing Officer
PNO: Principal Nursing Officer
SN: Staff Nurse
SNO: Senior Nursing Officer
SSN: Senior Staff Nurse
STROBE: Strengthening the Reporting of Observational Studies in Epidemiology
